# Cost‐effectiveness of alternative changes to a national blood collection service

**DOI:** 10.1111/tme.12537

**Published:** 2018-05-16

**Authors:** S. Willis, K. De Corte, J. A. Cairns, M. Zia Sadique, N. Hawkins, M. Pennington, G. Cho, D. J. Roberts, G. Miflin, R. Grieve

**Affiliations:** ^1^ Department of Health Services Research and Policy London School of Hygiene and Tropical Medicine London UK; ^2^ Institute of Health and Wellbeing University of Glasgow Glasgow UK; ^3^ Department of Health Services and Population Research King's College London London UK; ^4^ NHS Blood and Transplant London UK; ^5^ Radcliffe Department of Medicine and BRC Oxford Haematology Theme, University of Oxford John Radcliffe Hospital Oxford UK; ^6^ NIHR Blood and Transplant Research Unit in Donor Health and Genomics, Department of Public Health and Primary Care University of Cambridge Cambridge UK

**Keywords:** blood donation, cost‐effectiveness analysis, stated preferences

## Abstract

**Objectives:**

To evaluate the cost‐effectiveness of changing opening times, introducing a donor health report and reducing the minimum inter‐donation interval for donors attending static centres.

**Background:**

Evidence is required about the effect of changes to the blood collection service on costs and the frequency of donation.

**Methods/Materials:**

This study estimated the effect of changes to the blood collection service in England on the annual number of whole‐blood donations by current donors. We used donors' responses to a stated preference survey, donor registry data on donation frequency and deferral rates from the INTERVAL trial. Costs measured were those anticipated to differ between strategies. We reported the cost per additional unit of blood collected for each strategy versus current practice. Strategies with a cost per additional unit of whole blood less than £30 (an estimate of the current cost of collection) were judged likely to be cost‐effective.

**Results:**

In static donor centres, extending opening times to evenings and weekends provided an additional unit of whole blood at a cost of £23 and £29, respectively. Introducing a health report cost £130 per additional unit of blood collected. Although the strategy of reducing the minimum inter‐donation interval had the lowest cost per additional unit of blood collected (£10), this increased the rate of deferrals due to low haemoglobin (Hb).

**Conclusion:**

The introduction of a donor health report is unlikely to provide a sufficient increase in donation frequency to justify the additional costs. A more cost‐effective change is to extend opening hours for blood collection at static centres.

The World Health Organization (WHO) and the International Federation of Red Cross and Red Crescent Societies (IFRC) set out a shared global vision for a self‐sufficient blood supply by 2020 (WHO and IFRC, 2010). This framework for action called on blood supply agencies to encourage more frequent donation from current whole‐blood donors, such as by making blood donation more convenient. However, there is little evidence about the effect that changes to the blood collection service have on the frequency and costs of whole‐blood donation.

Blood supply agencies require evidence on the relative costs and effectiveness of alternative strategies, whether they are required to increase, decrease or maintain the current levels of whole blood supplied. In England, the overall demand for whole blood is falling, but there is increased demand for the universal blood type O negative (O−) as well as A negative (A−), B negative (B−) and rare blood subtypes more common in Black, Asian and minority ethnic (BAME) donors (e.g. Ro). A key challenge is to identify changes to the blood service that increase donation frequency for those donors whose blood type is in relatively high demand at low additional cost.

The Health Economics Modelling of alternative blood donation strategies (HEMO) study aimed to assess the cost‐effectiveness of strategies to maintain the blood supply in England (Grieve *et al.,* 2017, in press). The study estimated the frequency with which existing donors would be willing to donate whole blood following changes to the current blood collection service. This paper reports the essential features of the cost‐effectiveness analysis (CEA) and its implications for policymakers.

## MATERIALS AND METHOD

### *Strategies for the CEA*

In England, the NHS Blood and Transplantation (NHSBT) strategy emphasised the need to improve the donation experience for existing donors (NHS Blood and Transplant, 2015). The HEMO study therefore considered alternative service changes for increasing donation frequency for current whole‐blood donors; strategies to attract new donors were outside the study scope. The service changes of interest were identified through a review of NHSBT strategy documents, the results of market research, an informal review of relevant published literature, consultation with policymakers and insights from preliminary qualitative research with donors. The six strategies considered were the provision of a donor health report (at all blood collection venues), offering weekend and evening donation opportunities at either static centres or mobile sessions and reducing the minimum interval between donations for donors at static centres (see Table 1).

**Table 1:** Overview of the cost‐effectiveness analysis

Strategy	Target population	Attribute levels with status quo	Attribute levels with new strategy
Provision of health report for all donors	All donors who gave blood in last year.	Health report not provided	Health report provided
Weekend opening at static donor centres	All donors who gave blood in the last year at a static donor centre that is not routinely open at weekends.	Appointment availability *Every weekday Monday–Friday*	Every day: Monday–Sunday
Weekday evening opening at static donor centres	All donors who gave blood in the last year at a static donor centre that did not remain open until 20·00 on weekdays.	Current opening hours	Opening hours 09·00–20·00
Weekend opening of mobile sessions	All donors who gave blood in the last year at a mobile session that is not routinely open at weekends.	Appointment availability *1 day every 2 months: Monday–Friday*	Appointment availability *1 day every 2 months: Saturday or Sunday*
Weekday evening opening of mobile sessions	All donors who gave blood in the last year at a mobile sessions that is not routinely open until 20·00 on weekdays.	Current opening hours	Opening hours 14·00–20·00
Shorter minimum interval between donations for both men and women	All donors who gave blood in the last year at a static donor centre.	Maximum number of donations: Males four times per year and Females three times per year	Maximum number of donations: Males six times per year; Females four times per year

Each strategy involved a single change to the blood collection service compared to the current service experienced by whole‐blood donors. The strategies are not mutually exclusive and are not ‘scalable’ to the same degree, so we made a series of pairwise comparisons for each potential change compared to the current service provision. A 1‐year time horizon was adopted as the longer‐term demand for blood is unknown, and the shorter‐term effects of the alternative strategies on the volume and type of blood collected were considered more relevant for future policy.

### *Health report*

Several European blood supply agencies provide information about donors' own health to incentivise blood donation (Marantidou *et al.,* 2007). A health report provides donors with information about their own health from data routinely collected during blood donation, and sometimes from additional tests. The policy is controversial; blood supply agencies are not providers of healthcare, and evidence of its effectiveness in increasing the blood supply is mixed (Goette *et al.,* 2009). The HEMO study defined a health report as information provided to a donor on their blood pressure taken prior to donation and a cholesterol test taken from the blood sample after each donation. The analysis excluded any longer‐term sequelae following the health report.

### *Session opening times*

Holding sessions at evenings and weekends may make blood donation more convenient and increase donation frequency. To investigate realistic changes to opening times at static centres, we assumed that providing sessions at weekends or during weekday evenings would be *additional* to those provided during current opening hours. For mobile sessions, it was more realistic to assume that weekend or weekday evening sessions would *substitute* daytime sessions. In 2016, 86% of blood donations were made at a mobile session. In total, 23 000 mobile sessions were held in England for whole‐blood donation, of which only 10 % were open until 20:00 and only 4 % at weekends. Donors can also visit 1 of 24 permanent static centres where blood collection is offered in the same venue several days a week. Of the static donor centres, 15 were routinely open at weekends in 2016, and 5 offered sessions until 20:00 on weekday evenings.

### *Inter‐donation interval*

INTERVAL, a large multicentre, randomised, controlled trial, provided evidence on whether reducing inter‐donation intervals in all static centres in England would increase donation frequency without compromising donor safety (Di Angelantonio *et al.,* 2017; Moore *et al*. 2014). The trial reported that donors randomised to the shorter minimum donation interval (8 weeks for men, 10 weeks for women) successfully donated more whole blood on average compared to those randomised to the current minimum donation intervals (12 weeks for men, 16 weeks for women). Higher rates of deferral were recorded in the shorter donation interval randomised arms.

The HEMO study assessed the cost‐effectiveness of the shortest minimum inter‐donation interval adopted in the INTERVAL trial for men and women donating at static donor centres versus current minimum donation intervals.

### *Stated preference survey*

The CEA required predictions of the effects of alternative changes to the blood service on the frequency of blood donation. In England, these potential service changes have either not been implemented at all (e.g. the donor health report) or have only been implemented in some venues (e.g. weekend opening). We therefore needed to understand how donors might respond to these changes to the service without first experiencing them. Formal methods to elicit choices under hypothetical conditions, known as stated preferences, are used extensively in transport, environmental and health economics when information on actual choices, known as revealed preferences, are not available. We conducted a large stated preference survey of donors who had donated whole blood at least once in the previous year.

The stated preference survey was designed iteratively, incorporating the views of NHSBT policymakers and donors. The survey was revised following a large pilot study (De Corte *et al.,* 2016). The final survey included five attributes that described those characteristics of the blood service that were liable to be modified following proposed changes to the blood service. The chosen attributes were: donor travel time to the blood donation venue; the opening hours for blood collection; and the availability of appointments for blood donation, provision of a health report and the maximum number of whole‐blood donations permitted in a year. For each attribute, alternative levels were defined according to current and future service provision; e.g. for the health report attribute, two levels were defined according to whether or not a health report was provided.

**Figure 1:**
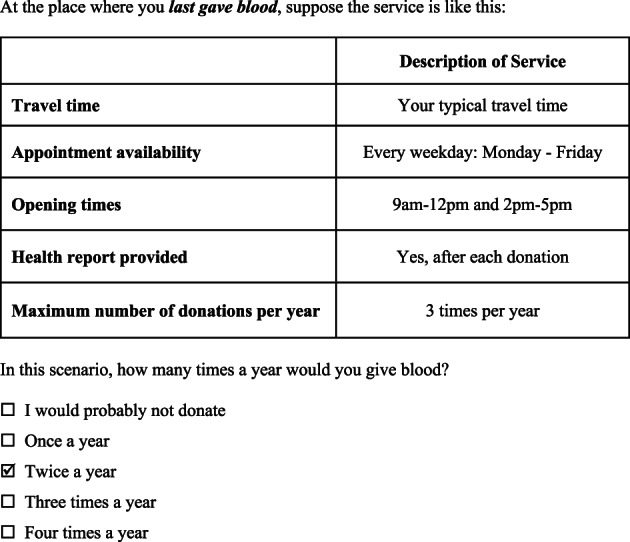
An example of a question from the stated preference survey.

Figure 1 presents an example question from the survey. Respondents were asked to state the frequency with which they would be willing to donate blood according to the alternative attributes and levels offered in different scenarios. We hypothesised that donors would state a higher frequency of donation if they were offered an incentive to donate, such as a health report, or if donation was made more convenient, e.g. by providing opportunities to donate at weekends or in the evening.

The survey received ethical approval from both NHS (reference 16/YH/0023) and LSHTM (reference 10 384) Research Ethics Committees. A total of 100 000 donors were randomly selected to be sent an email inviting them to take part in the online survey if they met the following inclusion criteria: 17–70 years old, donation of at least one unit of whole blood in the past 12 months, email address held by NHSBT and residence in mainland England. Donors were excluded if they had been temporarily suspended from donating (e.g. if they had recently had a tattoo) and if they had recently taken part in a routine survey or research study.

A total of 25 187 donors responded to the survey (25·2%). The donors who responded to the survey were somewhat different to the overall target population (Table 2), e.g. the proportion of donors over 60 years old was higher for the survey responders than the overall target population (21% vs 15%).

**Table 2:** Background characteristics of the population and respondents to the stated preference survey

		Donors who responded to the survey (*N* = 23 981)		All donors in March 2016 extract of PULSE database (*N* = 781 028) who had donated in the last 12 months	
		*N*	%	*N*	%
Age group	17–30	3 309	13·80	188 744	24·17
	31–45	5 774	24·08	205 505	26·31
	46–60	9 824	40·97	267 856	34·30
	60+	5 073	21·15	118 923	15·23
Blood type	High demand	2 472	10·31	111 948	14·33
	Standard demand	21 509	89·69	669 080	85·67
Ethnicity	White	22 339	93·15	724 880	92·81
	Black/mixed Black	201	0·84	8 315	1·06
	Asian/mixed Asian	562	2·34	21 727	2·78
	Other or not stated	879	3·67	26 106	3·34
‘Nursery’ donor	Yes	6 566	27·38	283 502	36·30
	No	17 415	72·62	497 526	63·70
Session type	Static centre	2 053	8·53	107 811	13·80
	Mobile session^1^	21 928	91·44	673 217	86·20
Number of donations in last 12 months	1	7 148	29·81	317 266	40·62
	2	8 063	33·62	245 984	31·49
	3	7 267	30·30	183 211	23·46
	4	1 454	6·06	29 460	3·77
	5	40	0·17	3 450	0·44
	6	9	0·04	1 657	0·21

1 A session is an organisational feature of NHSBT that can be understood as a single effort to collect blood on one particular day, by a particular team, in a particular location. For example, even if the same team collects blood at the same location for two consecutive days, this would be considered two sessions.

### *Target population*

The overall target population was all whole‐blood donors who had successfully given blood at least once in the year prior to March 2016 and who resided in mainland England (*N* = 781 028) (see Table 2). Although all donors were eligible for a health report after each donation, for the strategies that involved changes to opening times of the blood collection venues, the target population was limited to donors who last gave blood at a venue that was not already open at weekends and evenings. The target population for the strategy to reduce the minimum inter‐donation interval was limited to those donors whose last blood donation was at a static donor centre.

### *Predicting total volume of blood*

The responses to the stated preference survey were used to predict the average number of whole‐blood donations per year following the alternative changes to the blood service defined by each strategy. A major assumption is that individuals' responses to survey questions will predict their actual behaviour. We investigated whether this assumption was plausible and found that, on average, there was a small discrepancy between the donation frequency predicted from the survey responses and the actual donation frequency observed in the PULSE donor register (De Corte *et al.,* 2016; Grieve *et al.,* 2017, in press).

The data from the response to the survey were analysed to estimate the effect of potential service changes on the annual frequency of whole‐blood donation. As the response data were categorical and naturally ordered, an ordered logit model was chosen and included attributes from the stated preference survey as independent (exposure) variables (Greene, 2017). To allow for differences in observed characteristics between the survey responders and the overall target population, the model also included each of the characteristics listed in Table 2 as independent variables.

### *Adjusting for deferred donations*

If a donor's haemoglobin (Hb) levels are below 135 g L^−1^ for males or 125 g L^−1^ for females, blood collection NHSBT policy is that donation will be temporarily suspended, or deferred, for at least 6 months (or longer if Hb is particularly low). Donations can also be deferred due to other reasons, e.g. related to travel, medication, lifestyle or infection/illness. The INTERVAL trial reported that deferrals due to low Hb were higher for the patients randomised to reduced minimum interval (Di Angelantonio *et al.,* 2017). We used estimates from applying a logistic regression model to the trial data, which estimated the effect of changing the minimum interval on deferral rates and allowed for patient characteristics, to predict deferral rates per attendance according to the levels of those characteristics in the target population.

### *Difference in volume of blood collected between strategies*

The incremental effect of each strategy was calculated as the difference between the predicted mean volumes of blood before and after the proposed service change. The number of annual blood donation visits was calculated for each donor in the target population according to the donor's personal characteristics and the service‐level attributes that defined each donor's most recent experience of giving blood. We predicted the number of blood donation visits by combining the estimated coefficients from the ordered logit model applied to the survey response data with the characteristics of each donor in the target population. The predicted annual frequency of donation allowed for the estimated probability of deferral. The predicted annual mean number of units of blood donated per donor was then multiplied by the number of donors in the target population to calculate the annual total volume of blood collected across the service. Finally, the predictions were repeated after changing the attribute level associated with each proposed service change (see Table 1).

### *Costs*

Cost measurement was from the NHS and personal social services perspective recommended by The National Institute for Health and Care Excellence (NICE) (NICE methods guide, 2013). The costs included were those anticipated to differ between strategies, including additional collection and staff costs but not processing, marketing or fixed costs. Costs beyond 1 year were not considered. Three types of cost were included: the variable cost of collecting blood associated with each strategy (staff costs including unsocial hours premium, invitations, consumables), the costs of providing a health report, and the cost of deferrals.

### *Variable cost of blood collection*

The variable costs covered the cost of inviting donors, staff time and disposables. We assumed that processing costs were constant across strategies and that the service was scalable to any volume of blood collected. The cost measurement recognised differences in unit costs between mobile sessions and static donor centres and that, on average, mobile sessions were close to capacity (95%), whereas static centres were not (75%). The base case analysis therefore assumed that strategies which required more blood to be collected would require additional staff at mobile sessions but not in static centres, where additional collection within current opening times would be undertaken by existing staff. In both settings, the costs of staff employed at weekends and during evenings were calculated at appropriate additional rates (The NHS Staff Council, 2016).

### *Costs of providing a health report*

The health report costs assumed that the cholesterol test would be undertaken alongside others routinely undertaken at small additional cost (Czoski‐Murray *et al*. 2012; Department of Health 2008). We assumed that to measure blood pressure required an additional 1·5 min per donor. We assumed that 2% of tests would require a letter to advise clinical follow up.

### *Cost of deferrals*

The cost of deferrals included the time taken for donor carers to undertake a health screen and, where deferral was due to low Hb, a copper sulphate and HemoCue^®^ test (HemoCue^®^, Radiometer Medical ApS, Denmark). We assumed based on the INTERVAL trial data that 7% of these donors would be referred to their Primary Care Physician (when Hb is less than 125 g L^−1^ for men and 115 g L^−1^ for women) and then that healthcare costs would be incurred. These costs were assumed to include a GP appointment, a full blood count test and Serum ferritin test, iron supplements (50% of donors) and an outpatient appointment (10% donors). The accompanying unit costs were taken from published sources. (Curtis and Burns, 2016; Department of Health, 2016; Health‐Care Medical Equipment Group 2017; Joint Formulary Committee 2016; National Institute for Health and Care Excellence, 2015)

### *Cost‐effectiveness analysis*

The incremental cost per donor for each strategy compared to the status quo was calculated as an additional (difference in means) cost of collecting the additional (difference in means) volume of blood after the service change. We estimated the incremental cost per additional unit of whole blood collected overall and for subgroups of prime interest. These include five donor characteristics: age (17–30, 31–45, 46–60, 60 or over), high or standard demand blood types, ethnicity (White, Black/mixed Black, Asian/mixed Asian or Other/not stated), ‘nursery’ donor status (fewer than four lifetime donations) and the venue (static or mobile).

### *Interpretation of the threshold*

The threshold at which the health service in England is willing to pay to collect an extra unit of blood is unknown. The cost of a unit of blood for the NHS is around £120, half of which arises from the costs of collection, which differ across settings. For example, in England, the collection cost at a mobile session ranged from £23 to £60 per unit of whole blood (2015–2016), and sessions with relatively high cost per unit have since been closed. This implies that, in England, the willingness to pay for a unit of blood is likely to be around £30–£50, which we used to interpret our CEA.

### *Sensitivity analysis*

The cost‐effectiveness model was probabilistic; the uncertainty in the estimated incremental costs reflected the uncertainty in the volume of blood collected and associated resource use but not in the unit costs that were assumed fixed. We considered two sources of structural uncertainty: (i) the assumption about current operating capacity and (ii) the statistical model used to predict volume of blood. We recognised that static donor centres could require additional staff time to collect extra units of blood, and this increased the unit cost to £26·49 (£9·41 in the base case). This sensitivity analysis is not relevant for strategies two and three where additional staff costs are already included in the base case as these strategies represent the extension of current opening hours. We also considered alternative predictive models using a two‐part model and gamma model, rather than the ordered logit model used in the base case analysis.

## RESULTS

The effect of each change to the blood service on the average number of whole‐blood donations per donor per year are reported in Table 3 for each target population. The results show that donors would be willing to donate whole blood more frequently following each of the service changes. The largest predicted increase in average annual donation frequency was following strategies to reduce the minimum inter‐donation interval and to introduce weekend opening at static centres (annual increases of 0·71 and 0·49 donations per donor, respectively). Introducing a health report and providing mobile sessions in the evenings led to small increases in predicted donation frequency (0·1 and 0·03 per donor per year, respectively).

**Table 3:** Predicted deferral rates and adjusted annual donation frequency

Strategy		Average annual visits predicted per donor	Average annual number of low Hb deferrals per donor	Average annual number of other deferrals per donor	Deferral‐adjusted number donations per donor
Health report	Status quo^1^	2·595	0·092	0·151	2·362
	With health report	2·704	0·096	0·157	2·462
	Difference	0·109	0·004	0·006	0·100
Weekend opening of static centres	Status quo	2·604	0·092	0·150	2·374
	With weekend opening	3·142	0·112	0·181	2·864
	Difference	0·538	0·019	0·031	0·489
Evening opening of static centres	Status quo	2·779	0·099	0·160	2·534
	With evening opening	3·229	0·115	0·185	2·942
	Difference	0·45	0·016	0·026	0·408
Weekend opening of mobile sessions	Status quo	2·564	0·091	0·149	2·333
	With weekend opening	2·599	0·092	0·151	2·363
	Difference	0·035	0·001	0·002	0·03
Evening opening of mobile sessions	Status quo	2·518	0·089	0·146	2·291
	With evening opening	2·744	0·097	0·160	2·49
	Difference	0·226	0·008	0·013	0·199
Reduce minimum inter‐donation interval at static centres	Status quo	2·804	0·100	0·161	2·557
	Shorter inter‐donation interval	3·586	0·128	0·206	3·271
	Difference	0·782	0·028	0·045	0·714

1 Status quo refers to current blood service provision. The average annual visits predicted differs for the status quo comparator across the strategies because the relevant target population is not the same, as detailed in Table 1.

For each strategy compared to current practice, we report the incremental (difference in means) volume of blood collected, incremental costs and incremental cost per additional unit of blood for the relevant target population (Table 4). Although each service change was predicted to lead to additional donations of whole blood, this also led to additional costs, with the incremental cost per donor per year ranging from £3·16 to £18·12. These additional costs were for the variable cost of collecting the additional blood yield per donor. Aside from the introduction of the health report, these higher average costs were almost exclusively for the costs of collection *per se*.

**Table 4:** Incremental cost‐effectiveness of each strategy compared to the current blood service provision (mean values across 10 000 simulations)

Strategy	Number of donors affected	Incremental blood yield, units all blood types (nearest thousand)	Incremental volumeof blood per donor per year, units of blood	Incremental cost, £ GBP	Incremental cost per additional unit of blood, £ GBP
Reduce minimum donation interval	107 811	73 000	0·678	6·71	10
Evening opening of static donor centres	99 312	45 000	0·455	10·46	23
Weekend opening of static donor centres	60 640	31 000	0·519	15·21	29
Evening opening of mobile sessions	582 910	282 000	0·484	18·12	37
Weekend opening of mobile sessions	646 898	45 000	0·07	3·16	45
Health report	781 028	88 000	0·113	15·33	136

Table 4 ranks the strategies in order of their cost‐effectiveness. The strategy to reduce the minimum donation interval was predicted to provide additional units of whole blood at the lowest additional cost per unit, followed by the strategies of extending opening times for blood collection at static centres. The strategy to substitute mobile weekday sessions with sessions held at weekends had the lowest additional cost (£3·16) but the smallest predicted increase in blood donation, and was unlikely to be cost‐effective. At a cost of £136 per additional unit of blood, the introduction of the health report was very unlikely to be cost‐effective.

The main subgroup analysis was for donors with ‘high‐demand’ blood types and is reported in Table 5. The results were broadly similar in that the strategies with relatively low costs per additional unit of blood donated were the reduction of the minimum inter‐donation interval or weekend or evening opening for collection in static centres. The results of the other subgroup analyses revealed some differences in relative preferences for alternative service changes according to donors' characteristics; in particular, donors of Black, mixed Black, Asian and mixed Asian ethnicities were predicted to donate more frequently than donors of other ethnicities when offered the health report. However, the additional costs of the health report were such that the cost per additional unit of blood donated remained relatively high for this strategy (on average, £69 for Black/mixed Black donors and £102 for Asian/mixed Asian donors, compared to £136 for all donors). The cost‐effectiveness results of other strategies were very similar across all the subgroups considered.

**Table 5:** Base case results for donors with ‘high‐demand’ blood types

Strategy		Annual cost per donor (£GBP)	Total annual cost (all donors), 000 s (£GBP)	Total units (all) blood collected, 000 s	Incremental cost per additional unit blood (£GBP)
Health report	Status quo	21·57	2414	264	NA
	With health report	36·83	4123	276	NA
	Difference	15·27	1709	11	152
Weekend opening of static centres	Status quo	23·34	186	19	NA
	With weekend opening	37·63	300	23	NA
	Difference	14·29	114	4	29
Evening opening of static centres	Status quo	24·91	321	33	NA
	With evening opening	34·25	441	38	NA
	Difference	9·34	120	5	23
Weekend opening of mobile sessions	Status quo	21·02	1981	220	NA
	With weekend opening	22·37	2108	223	NA
	Difference	1·35	127	3	45
Evening opening of mobile sessions	Status quo	20·64	1756	195	NA
	With evening opening	28·1	2390	212	NA
	Difference	7·46	635	17	37
Reduce minimum inter‐donation interval at static centres	Status quo	25·13	349	35	NA
	Shorter inter‐donation interval	32·16	446	45	NA
	Difference	7·02	98	10	10

NA, not applicable.

In the scenario where staff costs were included in the variable cost per unit of blood for strategies one and six, the cost per additional unit of blood for these strategies increased to £27 (reduced interval) and £139 (health report). In this scenario, evening opening hours at donor centres was ranked the most cost‐effective strategy at £23 per additional unit of blood collected. When other analytical models were used for the analysis of the survey data, the ranking of strategies did not change compared to the base case.

## DISCUSSION

This analysis found that strategies that improve donation opportunities at static donor centres offered better value for money than the introduction of the health report or moving mobile sessions to weekends or evenings. The cost of opening static centres on weekday evenings or at weekends fell below £30 per additional unit of blood collected. These results were robust to the choice of model used to predict donation frequencies from the survey data and were similar across donor subgroups, including the subgroup of prime policy interest, those donors with ‘high‐demand’ blood types. These findings directly relate to the blood service in England and other public‐funded blood services required to increase the volume of particular types of blood and add to the limited literature on the cost‐effectiveness of alternative changes to a blood collection service (Van Der Pol & Cairns, 1998; Van Der Pol *et al.,* 2000; Varney & Guest, 2003; Dixon *et al.,* 2005; Pereira, 2006; Rautonen, 2007; Katsaliaki, 2008; Lowalekar & Ravichandran, 2010; Abraham & Sunday, 2012; Beliën & Forcé, 2012; Williamson & Devine, 2013).

Reducing the minimum interval between donations at static donor centre was the most cost‐effective strategy at £10 per additional unit of blood collected, but concerns remain about ‘rolling out’ a strategy of reducing the minimum interval for all donors. The INTERVAL trial reported lower levels of Hb for some donors over the 2‐year follow‐up period (Di Angelantonio *et al.,* 2017). Although our analysis did include the short‐term costs related to Hb deferral, the longer‐term impact of more Hb‐related deferrals on donor retention, and hence the long‐term cost‐effectiveness of this strategy, is unknown. The next most cost‐effective strategies, the opening of donor centres at weekends and evenings, may therefore make more efficient use of scare blood service resources, particularly if there is little capacity within the system to collect additional units of blood.

Not all strategies are scalable to the same degree. Strategies to improve opportunities to give blood for donors at static centres could yield between 60 000 and 100 000 units. To support the collection of additional blood in this quantity, staff would need to be redeployed from other sessions. Alternatively, the strategies could be implemented so that collection of high‐demand blood types is substituted for other blood types. If the extra collection of blood was limited to donors with high‐demand blood types, this would imply around 10 000 additional units of whole blood collected – which is much feasible within current staffing constraints. The results from our survey suggest that donors' preferences would be to donate these additional units of blood at more convenient times, namely, during the evenings and at weekends, which would also be relatively cost‐effective.

This analysis suffered from three main limitations. Firstly, despite the relatively high response rate for an online survey of the public, it is unclear whether the preferences of our survey responders are representative of the preferences of all recent donors. Although the ordered logit model did adjust for differences in measured characteristics between the sample and the target population, there may be differences in unobservable characteristics between the settings. However, there is no reason to suspect this would bias the CEA in favour of a particular strategy. Secondly, we did not consider the alternative strategies to recruit new donors, nor the effect beyond 1 year on the retention of existing donors. Thirdly, we did not include direct costs to donors, such as travel expenses. These costs may differ by strategy, but taking a wider societal perspective would also require that the increased utility for donors from the act of blood donation itself be included in the analysis.

The findings from the HEMO study are relevant to publicly funded blood supply agencies worldwide as they can be interpreted according to whether the objective is to increase or maintain the supply of particular blood types or for whole blood overall. Although costs and donor preferences are likely to differ between settings, this paper shows how large‐scale surveys of donors' preferences can generate the required information about alternative changes to a blood service to guide future policy.

Methods to define and analyse the impact of possible changes to blood collection from a health economic perspective are likely to become increasingly relevant to blood services faced with growing pressures. They offer a way forward in the attempt to balance the sometimes conflicting but insistent demands of economic efficiency, flexibility to accommodate short‐ and medium‐term fluctuations in demand and the need to reach different sections of the community.

## CONCLUSION

We found that moving mobile sessions to the weekend or providing health reports did not provide sufficient increases in the predicted donation frequency to justify the additional costs. Reducing the minimum inter‐donation interval increased volumes of blood donation at low costs in the short term, but the observed increase in Hb‐related deferrals over 2 years, may imply that this strategy is not cost‐effective in the longer term. Extending the opening hours of static donor centres is in line with donor preferences and provides a relatively cost‐effective way of providing additional units of blood, particularly blood types that are in high demand.

## CONFLICTS OF INTEREST

G. C., D. R., G. M. are all employees of NHS Blood and Transplant.

There are no other conflicts of interest to declare.
